# A Correlation-Based Anomaly Detection Model for Wireless Body Area Networks Using Convolutional Long Short-Term Memory Neural Network

**DOI:** 10.3390/s22051951

**Published:** 2022-03-02

**Authors:** Albatul Albattah, Murad A. Rassam

**Affiliations:** 1 Department of Information Technology, College of Computer, Qassim University, Buraidah 52571, Saudi Arabia; 411207333@qu.edu.sa; 2 Faculty of Engineering and Information Technology, Taiz University, Taiz 6803, Yemen

**Keywords:** anomaly detection, wireless body area networks, spatiotemporal correlation, convolutional neural networks, long short-term memory, deep learning

## Abstract

As the Internet of Healthcare Things (IoHT) concept emerges today, Wireless Body Area Networks (WBAN) constitute one of the most prominent technologies for improving healthcare services. WBANs are made up of tiny devices that can effectively enhance patient quality of life by collecting and monitoring physiological data and sending it to healthcare givers to assess the criticality of a patient and act accordingly. The collected data must be reliable and correct, and represent the real context to facilitate right and prompt decisions by healthcare personnel. Anomaly detection becomes a field of interest to ensure the reliability of collected data by detecting malicious data patterns that result due to various reasons such as sensor faults, error readings and possible malicious activities. Various anomaly detection solutions have been proposed for WBAN. However, existing detection approaches, which are mostly based on statistical and machine learning techniques, become ineffective in dealing with big data streams and novel context anomalous patterns in WBAN. Therefore, this paper proposed a model that employs the correlations that exist in the different physiological data attributes with the ability of the hybrid Convolutional Long Short-Term Memory (ConvLSTM) techniques to detect both simple point anomalies as well as contextual anomalies in the big data stream of WBAN. Experimental evaluations revealed that an average of 98% of F1-measure and 99% accuracy were reported by the proposed model on different subjects of the datasets compared to 64% achieved by both CNN and LSTM separately.

## 1. Introduction

The accelerated development of the Internet of Things (IoT) has attracted attention from stakeholders all over the world due to the combination of the physical world with the virtual world through the Internet for communication and data sharing. IoT has been defined as an interrelated system of mechanical and digital machines, computing devices and objects that is capable of transmitting data over a network without involving human-to-human or human-to-machine interaction. IoT becomes more prevalent every day in many life aspects such as industrial sectors, financial sectors, and healthcare sectors [1].

In healthcare, IoT has improved the quality of care provided to patients. Indeed, people can lead more comfortable lives as it guarantees their health and safety through continuity monitoring. In addition, it supports a wide range of applications, from implantable medical implants to Wireless Body Area Networks (WBAN). WBAN is composed of tiny devices that are considered the most promising technologies for improving healthcare services. These devices have enabled remote monitoring to enhance the overall quality of care provided to patients in remote areas or medical facilities [2,3]. 

Despite the merits, WBANs are vulnerable to external attacks as sensor data are collected from various locations and people. People with malicious intent may compromise the sensors and insert malicious data that constitute anomalous readings, leading to incorrect diagnoses and inappropriate medication for patients and substantial financial losses for any organizations that adapt the healthcare system [4,5]. Anomalies have emerged as a serious issue in healthcare systems. These anomalies may also result from faulty devices and erroneous readings of these devices. 

Machine learning and statistical techniques have been used to detect anomalies in systems in the past few years. Various researchers have studied the use of these techniques to detect anomalies in healthcare systems and their findings support their effectiveness as in [6,7]. However, despite their success, more research is needed to promote their improvement concerning the speed of detection, type of anomalies, the correlation that exists in the collected attributes, and dealing with big data. 

In this light, this paper proposes an anomaly detection model that exploits the correlation that exists in measured attributes of WBAN sensors and uses the hybrid ConvLSTM deep learning technique. This model aims to detect anomalous data in WBAN and consider the requirements of the learning process to identify anomalous behavior and provide a reliable system against sensor faults and anomalous activities with an understanding of the factors that impact patients and healthcare organizations. More specifically, it helps to ensure higher detection accuracy and fewer error rates by exploiting the multivariate spatiotemporal correlation between physiological data in WBAN. The contributions of this paper are summarized as follows:

(1) Developing a method that can benefit from the spatiotemporal correlation among physiological data and the contextual data to choose the most appropriate anomaly detection strategy under a given condition (normal and abnormal ranges for physiological data).
(2) Classifying the physiological data based on point anomaly (using the dynamic thresholds) and contextual anomaly (using anomaly score).
(3) Developing an anomaly detection model based on the ConvLSTM deep learning technique to detect both point and contextual anomalies. The proposed model fits big data requirements and time constraints that are important features of the WBAN.

The rest of this paper follows the following structure: Section 2 explores related works to anomalous detection systems in WBAN. Section 3 describes the design of the proposed model and its components. Section 4 presents the experimental results and analysis. Section 5 reports a comparative analysis with existing models while Section 6 concludes the paper and provides future research directions.

## 2. Related Works 

Anomaly detection receives more attention in the (IoT) domain, especially for healthcare systems that generate massive data from WBAN. Many anomalies detection models have been proposed for WBAN in the literature based on different mechanisms and are analyzed in the subsequent paragraphs.

In [3], the study proposed an anomaly detection approach to evaluate the difference between actual sensed data and predicted values that depend on historical measurements. The approach was then applied to real physiological healthcare data. Experimental results showed the effectiveness of the approach by achieving low false-positive rates and high detection rates. 

Using a Markov-based chain model, another study [6], evaluated the reliability of WBAN by detecting the anomalies, patient health status, hardware failure rate, and transient fault correction method. The study proposed a metric for Mean Time to Failure (MTTF) that provided better performance in analyzing the reliability in specification and anomaly detection for WBANs. The results showed a 95% detection rate and a lower MTTF value of 43.01 s. Although the study showed a high detection rate as a reliability metric, the reliability metric is not enough to cope with some types of anomalies (i.e., fault measurements and abnormal readings).

In [7], the study suggested a dynamic threshold approach to detect the sensor anomaly and differentiate between true and false alarms effectively. This approach used a correlation method to extract the features and estimated the sensor values using random forest algorithms. The proposed approach was used to analyze past historical physiological data and compare it with predicted sensed values. The error value was measured using a dynamic threshold to identify the false and true alarms. The results showed a high detection rate and a low false-positive rate. However, this approach exploits only the spatial correlation between two sensors and ignored the temporal correlation. Although the random forest achieved perfect results, its complexity invokes long training periods.

In [8], an approach was proposed to detect changes that occurred in data collected by WBAN based on the Kalman filter algorithm. Authors claimed that this approach can automatically detect any physiological change that occurred in WBAN. Nevertheless, the Kalman filter has some downsides such as a larger computational complexity to obtain the best results. In [9], the study suggested a framework for anomalous sensor data detection. The framework was based on Hadoop Map Reduce-based parallel fuzzy clustering and data compression. The results showed that the proposed framework achieved high accuracy with fewer false alarms, which obtained an accuracy between 97% and 98%. This study used a parametric statistical approach that involves high computational complexity.

In [10], the study proposed a framework that combined regression techniques and random forest algorithm to detect anomalies in WBAN. This framework considered both temporal and spatial correlations to detect anomalies. An accuracy of 96% was reported for the proposed framework. However, the combination of random forest with regression is not able to detect new forms of anomalies.

In [11], the study conducted an approach for detecting abnormalities changes such as modifications, forgery, and insertions that occur in electrocardiogram (ECG) data. The approach utilized the Markov model with different window sizes of abnormalities data (5% and 10%). The results reported 99.8% of true negatives with the 5% and 98.7% of true negatives with the 10% windows. This study just used one type of data for detecting abnormality and emergencies. Despite the good performance of this model in terms of time execution, it still has some limitations in terms of memory.

In [12], researchers proposed a new approach to detect anomalies in WBAN based on Gaussian regression and majority voting. The proposed approach created a system that can distinguish between real medical conditions and false alarms by using a real dataset. Reported findings showed that this approach was effective in terms of high detection rate and low false-positive rate. However, there were some limitations to this approach such as high computation complexity, high false alarm, and bulky data samples.

In [13], the study suggested enhancing anomaly detection by adding a correlation algorithm for various body sensor types. The suggested algorithm utilized thresholds to detect anomalies. The results showed an improvement in various intersections between analyzed medical signals. However, the study only considered one type of correlation that exploits the spatial relationship between sensors and ignore the temporal correlation at each sensor reading. Two subsequent studies in [14,15] measured the faultiness of the sensors that cause high false alarms in healthcare systems. Both studies used dynamic sliding window and weighted moving average to detect the abnormal sensor faulty measurements. However, using the weighted moving average approach often overlooks the complicated relationships that exist in the data.

In [16], the authors developed an anomaly detection method for WBAN to discard false alarms caused by faulty measurements. The method used the spatiotemporal correlation and a game-theoretic technique. In addition, it applied Mahalanobis distance at the Local Processing Unit (LPU) for multivariate analysis. The proposed method proved superior effectiveness in achieving low false alarm rates with high detection accuracy. A possible weakness of the game-theoretic technique is that it cannot deal with new forms of anomalies.

In [17], the study developed an anomaly detection model for medical WSN. The model aimed to achieve a low false-positive rate with a high quality of detection. Moreover, this model used a decision tree, linear regression and threshold biasing and was then tested using a real physiological dataset. The empirical results of the study showed a high performance where the false-positive rate was 4.5%. However, machine learning algorithms impact inefficient performance when dealing with complex big data generated by medical WSN. The authors in [18] proposed a shapelet-base (SH-BASE) approach to detect anomalies. The experiment results revealed that SH-BASE achieved average performance in sensitivity and accuracy. However, the drawbacks of this approach are poor generalization capability and a high computational burden.

In [19], The work integrated the artificial neural network with ensemble linear regression to detect anomalies in WBAN. This work helped to create distinction in anomalous data by classifying the physiological data and then applying regression to identify the anomalous data. Experiments revealed that the proposed approach was able to reduce false alarms by 4.2%. Moreover, linear regression has some certain limitations such as that it cannot give feedback and sensitivity with anomalies.

In [20], the research proposed an anomaly detection system for WBAN based on the data sampling approach with Modified Cumulative Sum (MCUSUM). The sampling method was applied to increase the speed of detecting anomalies, while the MCUSUM algorithm was applied to accurately detect anomalies. The results showed that the proposed approach provided the lowest execution time and high energy efficiency of the sensors. However, the approach cannot detect random anomalies in various physiological parameters.

In [21], the authors recommended an approach founded on the Markov model to detect anomalies in WBANs. The approach used forecasting data to lower the amount of energy consumed in healthcare facilities. This approach aimed to determine whether a system is operating normally or not. Moreover, when the system is operating abnormally, the approach determines whether the anomalies are psychological. Experimental results revealed that the approach had a low false alarm rate of 5.2% and achieved high detection accuracy. The demerit of the approach came from the fact that Markov models are inappropriate regarding memory and computing time.

In [22], authors evaluated five machine learning approaches (random forests, local outlier factor, isolation forests, support vector machines, and K-Nearest neighbors) in their ability to detect anomalies in heart rate data. The best results were reported for random forests and local outlier factor approaches, where random forests achieved 100% and the local outlier factor 96.89% of correct rejection rate. Nevertheless, applying machine learning algorithms may have drawbacks since the output depends on the input set to predict; therefore, these algorithms are not effective when dealing with a new pattern of anomalies.

Recently, in [23], the authors proposed an anomaly detection approach for wearable computing devices (WCDs) to measure the faulty and malicious data that might endanger the patient’s life under monitoring. The approach used data classification methods for four classification algorithms: FURIA, ID3, J48, and PRISM. The four algorithms have obtained different results such that the FURIA outperforms other algorithms with 95.87% of the true-positive rate and 0.5% of the false-positive rate compared to ID3 with 69.9%, J48 with 84.28%, and PRISM with 79.3% true-positive rate. 

In [24], the study conducted a lightweight anomaly detection (LWAD) framework for detecting anomalies in WBAN. The framework was based on distance correlation with a statistical-based improvised dynamic sliding window algorithm for efficient prediction in short-range. The validation of the framework was verified using three real-time datasets. The proposed LWAD obtained a high detection rate of 99.65% for dataset 1 (DS1), 98.75% for DS2, and 98.18% or DS3. The statistical techniques are faster and less complex, but cannot deal with the normal data distribution and the dynamic nature of WBAN.

Based on the above discussion of the existing works, the following subsections analyze them based on two aspects that are the anomaly-related factors, and the used techniques.

(a) Anomaly-Related Factors

Table 1 summarizes and presents an analysis of existing anomaly detection models for WBAN based on the anomaly type, correlation approach, threshold type, and the consideration of fault measurements. In terms of anomaly type, the point anomaly refers to utilizing individual attributes of the dataset, whereas multiple attributes are considered together in the contextual anomaly approaches. In terms of the correlation approach, temporal correlation corresponds to the readiness of a single node in time instants while the spatial correlation corresponds to the readings of nodes compared with their neighboring nodes [25]. Two types of thresholds are adopted that are dynamic and static. The dynamic threshold is the prediction of a sensor value based on some historical dataset measurements, while the static threshold is manually selected based on previous knowledge of the field [3,26]. Finally, when only one of the attributes is found to be anomalous, the measurement is considered faulty and is therefore known as fault measurement [16].

Looking at Table 1, the challenges in the existing techniques can be attributed to the following grounds:

- Most existing studies focused on detecting point anomalies in sensor readings to predict the next potential sensor value and compare it to the actual reading. Few studies considered the case of contextual anomaly between various sensor readings.
- The temporal correlations play an important role in anomaly detection as the various attributes of multi-variant data may show varying temporal correlations. In addition, the attributes are characterized by frequent changes in data distributions with time. Spatial correlations imply that the data values at a particular sensor node are related to the data samples of the neighboring sensors nodes. As shown in Table 1, most of the previous studies take the correlations separately, either temporal or spatial. The optimal anomaly detection technique must incorporate these correlations to achieve the following types of dependencies (between sensor node characteristics, sensor node readings are dependent on time and their history, and sensor node reading is dependent on its neighboring nodes).
- Some existing studies used static thresholds, which may not be sufficient for some reasons; the threshold value of the physiological data is different from one person to another, and it relies on the factors of the person’s lifestyle, age, and medical condition. Therefore, the static threshold will not efficiently work for health monitoring, due to the slow error rate computation and response time. In addition, it is not able to adapt to the continuous changes in medical environments and it requires much effort since it is a manual process. Hence, using dynamic thresholds will be the optimal choice because they calculate value based on the input data (physiological data). Consequently, the value will be appropriate and accurate with WBAN data.

(b) Techniques

Sensors are used in different healthcare monitoring applications, and the data are growing extremely. Therefore, several techniques have been proposed in the literature to improve the effectiveness of anomaly detection approaches in WBAN such as statistical or machine learning techniques. However, such techniques have certain limitations. For example, the statistical techniques cannot deal with the dynamic nature of WBAN and it serves to choose the appropriate threshold value for evaluation. In addition, the non-parametric statistical method is not suitable for real-time applications due to its computational burden. Many of the current techniques use machine learning algorithms such as decision tree, linear regression, artificial neural network, nearest neighbor and random forest. The use of machine learning may not be the preferred option in a sensitive domain such as healthcare that requires high accuracy and good performance. These algorithms have certain limitations in dealing with complex and big data, slow computation, and expecting new patterns of anomalies.

To conclude, existing studies have focused on designing techniques for anomaly detection based on correlation with statistical or machine learning, but these techniques are still insufficient to solve the issues. In this domain, detecting an anomaly in the healthcare system requires more effort to understand the nature and importance of the data attributes. Furthermore, a more accurate model that can deal with complex real scenarios is required. In opposition, deep learning is an excellent candidate for overcoming the constraints of the existing techniques mentioned above. Deep learning techniques are capable of learning the inherent data characteristics that distinguish a normal data point from an anomalous one. This approach identifies commonalities in the data and therefore facilitates the detection of anomalies. It is also considered a cost-effective approach for detecting abnormalities because it does not require annotated data for training the algorithms [27]. Furthermore, the architectures of deep learning models are dynamic, allowing them to adapt to new patterns. Therefore, it has the potential to outperform machine learning and statistical methods in dealing with huge amounts of data collected by sensors in WBAN.

## 3. Proposed Model

**Figure 1:**
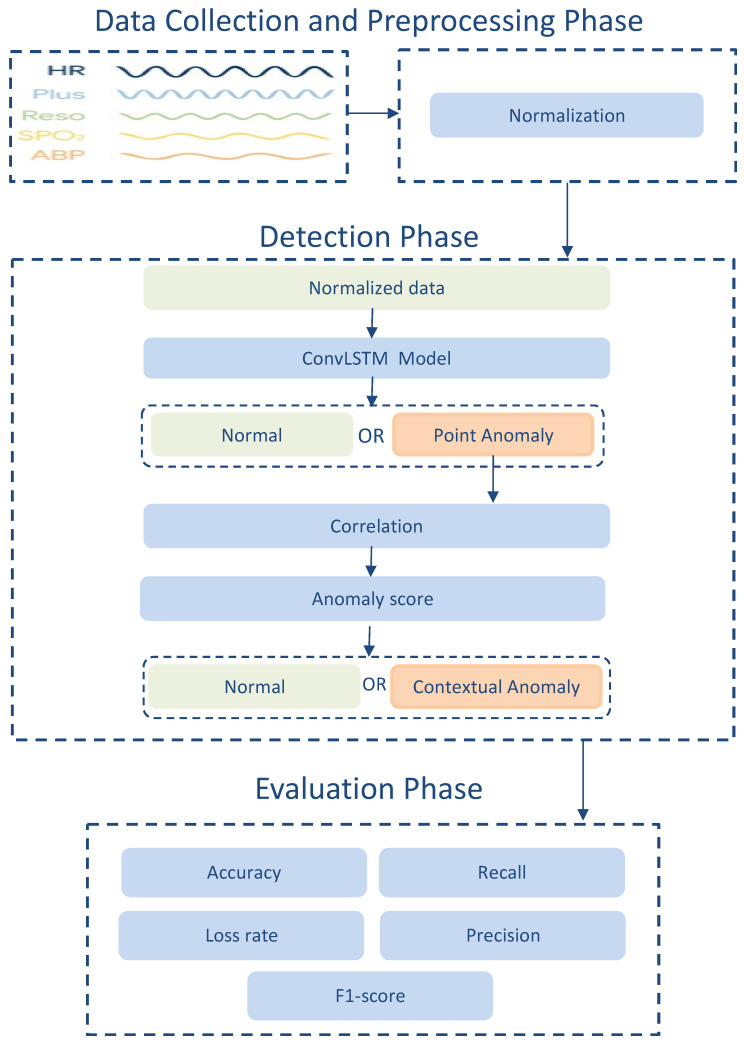
Proposed Mode.

The proposed model involves three phases: Data collection and preprocessing phase, detection phase, and evaluation phase. The data collection and pre-processing phase are used to collect the data by the different physiological sensors and clean the data before applying the detection model. The detection phase is to distinguish between the normal and anomalous data. This phase is divided into point anomaly detection and contextual anomaly detection. The evaluation phase is to test the performance of the model. Figure 1 shows the workflow of the proposed model. 

### 3.1. Data Collection and Pre-Processing Phase

**Figure 2:**
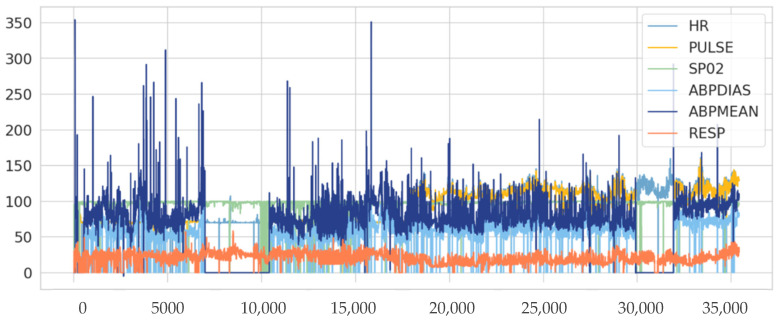
Sensors readings for Data Subject 1.

***Dataset***: The dataset used in this paper is Multiple Intelligent Monitoring in Intensive Care (MIMIC-I and II) [28], which contains detailed physiological data records recorded from over 90 ICU patients called subjects. Notably, most researchers such as [23,29,30,31] used the MIMIC dataset as a benchmark to test the viability of the proposed models. In this paper, we test the proposed model using four subjects (1, 2, 3, and 4). The dataset has 7 features. These features represent the patient’s clinical condition and include Heart Rate (HR), systolic Arterial Blood Pressure (ABPsys), diastolic Arterial Blood Pressure (ABPdias), Mean Arterial Blood Pressure (ABP-mean), Pulse, Temperature, Respiration Rate (RESP), and Oxygen Saturation (SPO2) with timesteps and date. Table 2 and Figure 2 present a sample of sensor readings for the dataset of Subject 1.

***Pre-processing***: Normalization is a method often used as part of data preprocessing and preparation for deep learning. Normalization aims to rearrange the values of numeric columns in the dataset to utilize a common scale, without deforming variations in the ranges of values or losing data. Moreover, the normalization is a demand of some algorithms to model the data accurately [32]. For this paper, each column in the dataset samples is normalized to be in a range between 0 and 1, using Equation (1).

(1) $$x\left(i\right)=\frac{x\left(i\right)-\bar{x}}{S\left(x\right)}$$

where x(i) is the dataset, $\bar{x}$ is one column in the dataset and S(x) is the number of the data sample.

### 3.2. Detection Phase

This phase aims to detect anomalous readings in WBAN using a deep learning approach. Deep learning has been used in many aspects of applications primarily due to its capability to automatically detect complicated features without having any field knowledge. This automatic feature learning capability makes the neural network a perfect candidate for time-series anomaly detection problems [33]. Therefore, the hybrid ConvLSTM technique is used in this paper to detect the anomaly readings in WBAN. In addition, the ConvLSTM is well in spatiotemporal relationships and both the input-to-state and state-to-state transitions [33]. Finally, it is robust to changes as compared to other neural networks and statistical models.

#### 3.2.1. Long Short-Term Memory (LSTM)

**Figure 3:**
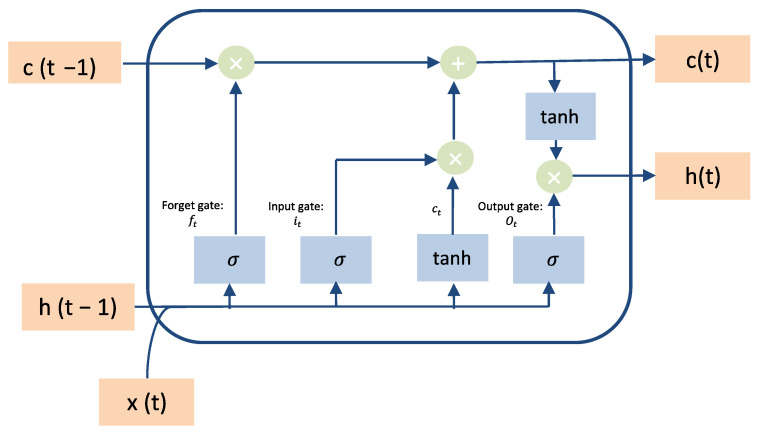
LSTM cell architecture.

Long Short-term Memory (LSTM) is a type of Recurrent Neural Network (RNN) that is utilized to overcome the palace of RNN. LSTMs are qualified to handle long-term dependencies via substituting the hidden layers of RNN with memory cells [34], as shown in Figure 3.

In LSTMs, there are various gate units such as output gate (o_t_), input gate (i_t_), forget gate (f_t_) with the activation function that is applied to LSTMs model and understands the behavior of temporal correlations [34].The LSTM cell can be defined mathematically in Equations (2)–(7) [32] Table 3 shows the shortcuts for the equations.

(2) $$f_{t}=\sigma \;(W_{f}\left[h_{t-1},X_{t}\right]+B_{f})$$

(3) $$i_{t}=\sigma \;\left(W_{i}\left[h_{t-1},X_{t}\right]+B_{i}\right)$$

(4) $$c\;{(}_{t-1})=\tanh\left(W_{c}*\left[h_{t-1},X_{t}\right]+B_{c}\right)$$

(5) $$c_{t}=c_{t-1}*f_{t}+i_{t}*{c^{\prime }}_{t}$$

(6) $$o_{t}=\sigma \;\left(W_{o}\left[h_{t-1},X_{t}\right]+B_{o}\right)$$

(7) $$h_{t}=o_{t}*\tanh\;\left(c_{t}\right)$$

#### 3.2.2. Convolutional LSTM (Conv-LSTM)

The hybrid deep learning model convolutional LSTM (ConvLSTM) integrates two architectures that are the convolutional and the LSTM techniques. ConvLSTM is a kind of recurrent neural network that captures spatial-temporal data [35]. The convolutional component captures the spatial area data and LSTMs exploits the temporal area data. However, the data in the form of time series from various sensors have a correlation with each other that depend on space and time. Thus, ConvLSTM can be used as a significant model for the time-series data anomaly detection problems [34].

**Figure 4:**
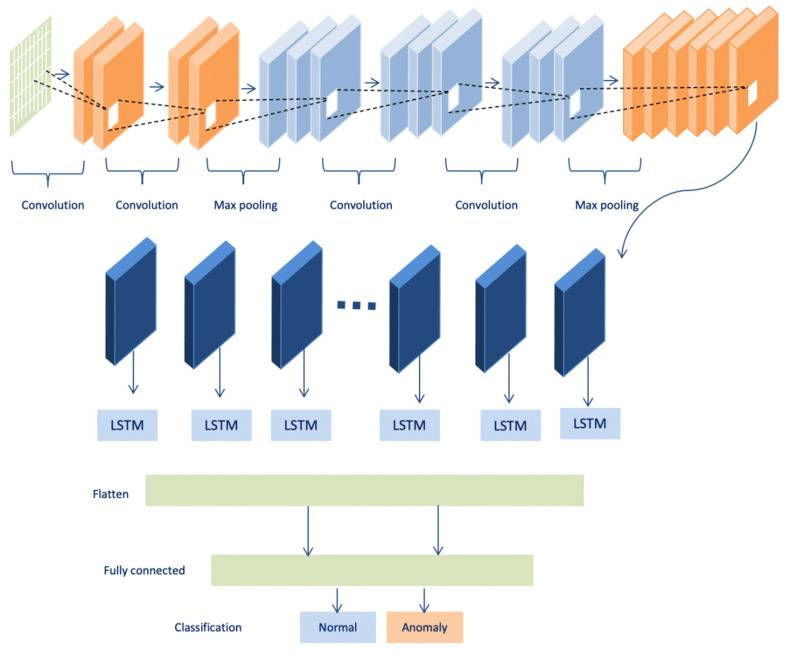
CNN-LSTM (ConvLSTM) Architecture.

The ConvLSTM determines the future state of a cell in the network via the inputs and past states of its local neighbors through which both the temporal and spatial correlations are captured and applied. This is achieved by replacing the matrix multiplication operation utilized in standard fully connected long short term memory (FC-LSTM) with convolution operation in the state-to-state and input-to-state transitions [35], as shown in Figure 4. The ConvLSTM contains several gates (input gate, forget gate, and output gate) and data flow can be expressed in Equations (8)–(13) with all the variables defined and described in Table 4. In the equations, “⊗ “ refers to the convolution operation, and “⨀” refers to the Hadamard product. σ is a sigmoid function utilized as the activation function used to the weighted sum of the inputs of each gate [36]. Table 4 shows the shortcuts for the equations.

(8) $$f_{t}=\sigma \left(W_{\mathrm{xf}}⊗X_{t}+W_{\mathrm{hf}}⊗H_{t-1}+W_{\mathrm{cf}}⊙C_{t-1}+B_{f}\right)$$

(9) $$i_{t}=\sigma (W_{\mathrm{xi}}⊗X_{t}+W_{\mathrm{hi}}⊗H_{t-1}+W_{\mathrm{ci}}⊙C_{t-1}+B_{i})$$

(10) $${c^{\prime }}_{t}=\tanh\left(W_{\mathrm{xc}}⊗X_{t}+W_{\mathrm{hc}}⊗H_{t-1}+B_{c}\right)$$

(11) $$c_{t}=f_{t}⊙C_{t-1}+{c^{\prime }}_{t}$$

(12) $$o_{t}=\sigma (W_{\mathrm{xo}}⊗X_{t}+W_{\mathrm{ho}}⊗H_{t-1}+W_{\mathrm{co}}⊙C_{t}+B_{o})$$

(13) $$h_{t}=o_{t}⊙\tanh\left(c_{t}\right)$$

#### 3.2.3. Point Anomaly Detection 

**Figure 5:**
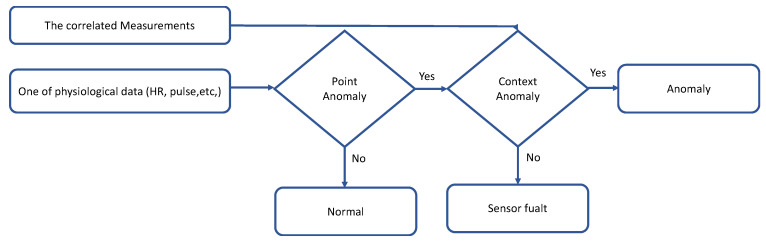
Process of calculating the point anomaly.

In this subphase, the data are classified based on the actual range of physiological data; for example, the normal range of HR will be between 60–100, and if values are higher or lower than the normal range this is considered an anomaly [23]. Table 5 describes the range classification of all physiological data, and the process is described in Figure 5.

##### Dynamic Threshold

To determine whether a sensor reading is point anomaly or not, the dynamic threshold is used. Each threshold value for physiological attribute values is unique and varies from subject to subject (it depends on some factors such as sex, age, and lifestyle). Moreover, the same subject’s threshold value can differ due to differences in the subject’s medical condition. Therefore, the static threshold value is not sufficient for a health monitoring system as it does not measure the accurate error value of the subject in different instances. The Mean Absolute Error (MAE) [7,37] is utilized here to calculate the dynamic threshold as shown in Equation (14).

(14) $$\mathrm{MAE}=\frac{\sum_{i=1}^{N}\left|x\left(t\right)-\bar{x}\left(t\right)\right|}{N}$$

where x(t) is the actual data, $\bar{x}\left(t\right)$ is the predicted data, and *n* is the total number of data instances.

##### Correlation Testing 

**Figure 6:**
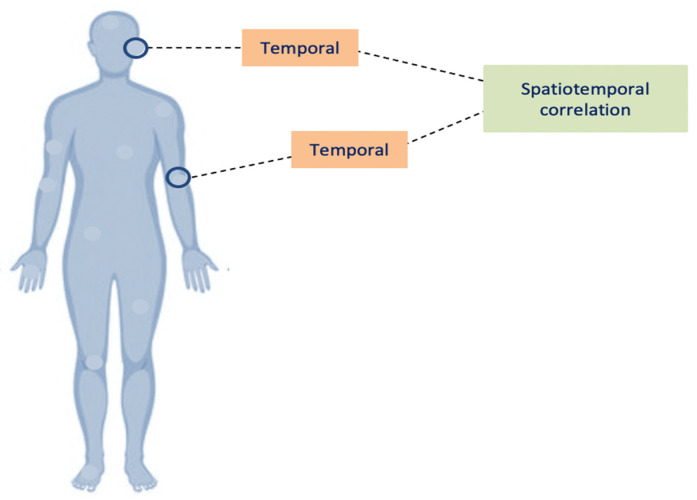
Concept for spatial and temporal correlation.

This subphase aims to test the relationship between the physiological data parameters. For example, consider two medical sensors: one for monitoring blood pressure and the other for measuring pulse. In general, when blood pressure rises, so does pulse rate. Thus, for a real medical condition, both sensor values must be abnormal; otherwise, if one sensor value is abnormal but the other is not, it is a sensor fault. As a result, the context of a sensor is taken into account to decrease the frequency of false alarms [29]. Two methods are used for measuring data relationships (temporal and spatial correlation). The temporal correlation at a single node location is caused by data value change over time. In contrast, the spatial correlation at a single node location is a result of a comparison with neighboring nodes. The combination between these two concepts is known as spatiotemporal correlation, which is detected by several node locations owing to variations in data value over time and place [25], as shown in Figure 6. Consequently, the proposed model in this paper uses both methods temporal and spatial to find contextual anomalies in physiological data based on the Pearson correlation coefficient [29]. The value of the correlation coefficient between various sensors is calculated and represented in the form of a correlation matrix (*C_n×n_*) given as Equation (15).

(15) $$\mathrm{Correlation}\;\mathrm{Matrix}\;(C_{n\times n})=\left[\begin{array}{ccc} Corr\left(y_{1}y_{1}\right)\;Corr\left(y_{1}y_{2}\right) & \cdots & Corr\left(y_{1}y_{n}\right)\\ Corr\left(y_{2}y_{1}\right)\;Corr\left(y_{2}y_{2}\right) & \cdots & Corr\left(y_{2}y_{n}\right)\\ \cdots & \cdots & \cdots \\ \cdots & \cdots & \cdots \\ Corr\left(y_{n}y_{1}\right)\;Corr\left(y_{n}y_{2}\right) & \cdots & Corr\left(y_{n}y_{n}\right) \end{array}\right]$$

The Pearson correlation coefficients are expressed in Equation (16).

(16) $$C=Corr\left(y_{1}y_{2}\right)=\frac{\sum y_{1}y_{2}-\frac{\sum y1\;\sum y2}{m}}{\sqrt{\left(\sum y_{1}{}^{2}-\frac{{\left(\sum y1\right)}^{2}}{m}\right)\left(\sum y_{2}{}^{2}-\frac{{\left(\sum y2\right)}^{2}}{n}\right)}}$$

#### 3.2.4. Contextual Anomaly Detection 

**Figure 7:**
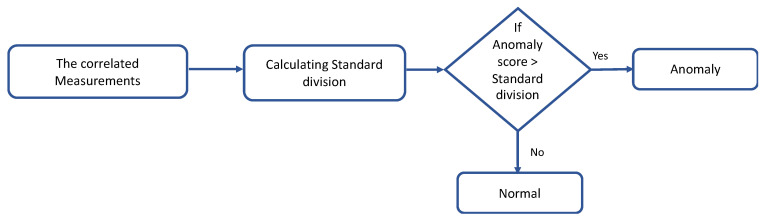
Process of calculation the contextual anomaly.

The values of physiological data may vary from one sensor to another depending on physiological conditions. Therefore, the physiological data in the sensor are considered an anomaly when compared with the other correlated data in another sensor. In this case, it is important to use the anomaly score method to reflect normal and anomalous physiological data [30]. The anomaly score is then compared with the standard deviation. The process of calculation is explained in Figure 7.

Let X(p) refer to the time series collected and correlated measurements of physiological data.

(17) $$X\left(p\right)=\left\{x_{1},x_{2},\ldots \ldots ,x_{5}\right\}$$

An anomaly score of physiological data measurement *X*(*p*) represents the deviation from the mean of the recent previous measurements [16]. The sensor Anomaly Score (AS) is given in Equations (18)–(19).

(18) $$\mathrm{Mean}=\frac{\sum X\left(p\right)}{N}$$

(19) $$\mathrm{Anomaly}\;\mathrm{Score}\;\left(\mathrm{AS}\right)=\frac{\sum |x-\mu |}{\sigma }$$

To label the current measurement as an anomaly, the AS value should be greater than the standard deviation of the past measurements [16]. The standard deviation (σ) is given in Equation (20).

(20) $$\sigma =\sqrt{\frac{\sum {|x-\mu |}^{2}}{N}}$$

where ∑ means “sum of”, *x* is a sensor value in the physiological data, μ is the mean of the physiological data, and N is the number of data instances. A comparison between AS with standard deviation is done using Equation (21).

(21) $$\{\begin{array}{c} \mathrm{AS}>\sigma \;\left(anomaly\right),\\ \mathrm{AS}<\sigma \;\left(normal\right) \end{array}$$

### 3.3. Evaluation Phase 

In this subphase, the proposed model performance is evaluated and compared with selected recent and best existing models. 

The classification models predict the class of each data instance, assigning a predicted label (positive or negative) to each sample [38]. 

Four parameters are used to measure the performance of the proposed model using the previous four categories, which are detection accuracy, recall, precision, and the F1-score.

The accuracy is a statistical assessment of how well a model predicts [39]; Equation (22) shows how the accuracy metric is determined:

(22) $$\mathrm{Accuracy}=\frac{\mathrm{TP}+\mathrm{TN}}{\mathrm{TP}+\mathrm{TN}+\mathrm{FP}+\mathrm{FN}}$$

Recall and precision are commonly used to assess the accuracy of a result [40], which are properly described as in Equations (23)–(25).

(23) $$\mathrm{Recall}=\frac{\mathrm{TP}}{\mathrm{TP}+\mathrm{FN}}$$

(24) $$\mathrm{Precision}=\frac{\mathrm{TP}}{\mathrm{TP}+\mathrm{FP}}$$

F1-score is a weighted average of recall and precision that is utilized when the data are unbalanced [41]; Equation (25) shows how the F1-score metric is determined.

(25) $$F1\_\mathrm{score}=2\cdot \frac{precision\;\cdot \;recall\;}{precision+recall}$$

## 4. Experiments and Results

In this section, the setup of the proposed model and the detailed results are presented.

### 4.1. Model Setup

This paper chooses the MIMIC dataset (2021) [28], a large-scale physiological data dataset described in Section 3.1 The proposed model was implemented in Python with Sklearn library and the assistance of other scientific computing libraries: Matplotlib, NumPy, and Scikit-learn to implement various tasks, such as preprocessing and model selection.

Adam optimizer was adopted as the optimization algorithm for the ConvLSTM technique. The ConvLSTM contains 4-layers network with 2 layers of dropout [ (filters = 64 in 2 layers), (kernel_size = 1), (padding = “same” in 2 layers), (activation function = “Relu”), (samples dimension of the 2-D tensor)]. The models are trained using a batch size of 30 with a different number of epochs. The dataset was split into training and testing datasets using a ratio of 70:30 for train and test partitions, respectively. 

### 4.2. Results and Analysis

In this subsection, the results of point anomaly detection and contextual anomaly detection are provided.

#### 4.2.1. Point Anomaly Detection Results and Analysis

**Figure 8:**
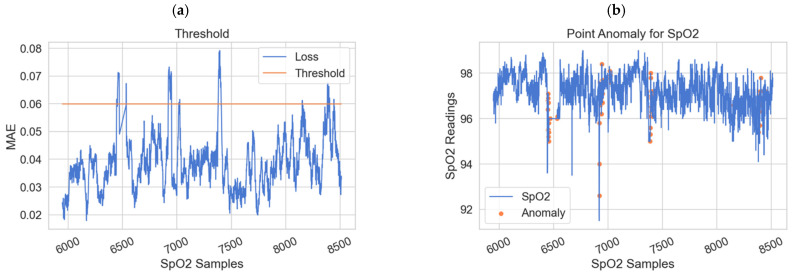

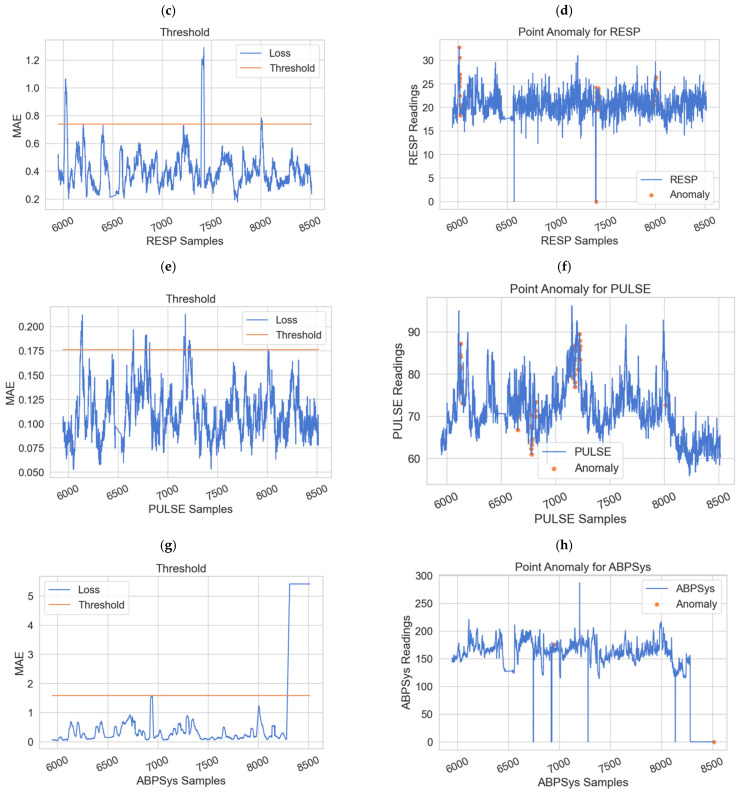

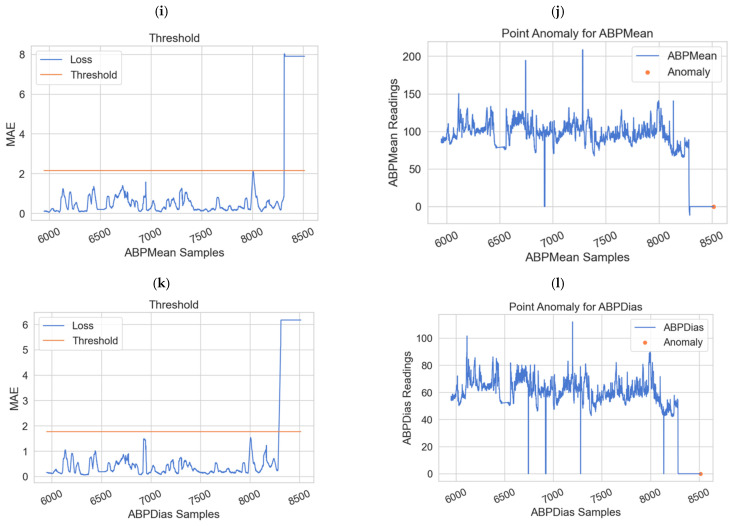
The value of the dynamic threshold and the respective point anomalies for various physiological sensors: (**a**) SPO2 threshold. (**b**) SPO2 point anomaly. (**c**) RESP threshold. (**d**) RESP point anomaly. (**e**) Pulse threshold. (**f**) Pulse point anomaly. (**g**) ABPSys threshold. (**h**) ABPSys point anomaly. (**i**) ABPMean threshold. (**j**) ABPMean point anomaly. (**k**) ABPDias threshold. (**l**) ABPDias point anomaly.

The physiological data reading is determined as a point anomaly if the classification value exceeds the dynamic threshold; otherwise, it is normal physiological data. The standard deviation of the MAE acts as a dynamic threshold based on the studies in [7,42]. Figure 8 shows the dynamic threshold for SPO2, RESP, Pulse, ABPSys, ABPMean, and ABPDias physiological sensors. As shown, the value of the dynamic threshold is different from one sensor to another. It further shows the point anomaly results for these sensors. If the values are larger or smaller than the normal ranges of these parameters, they will be considered anomalies. 

**Figure 9:**
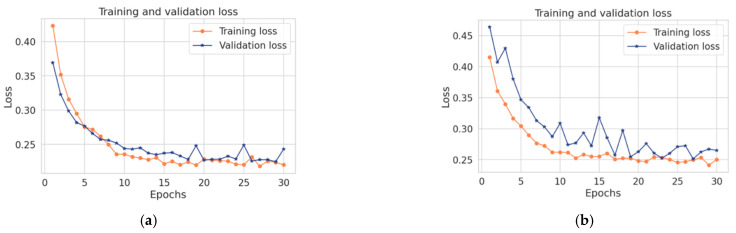

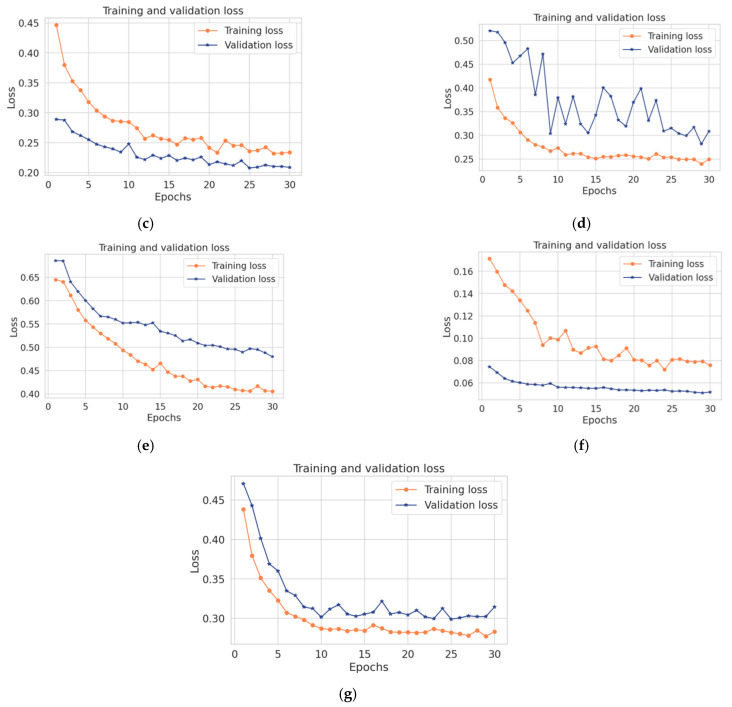
Loss rate for the various physiological sensors. (**a**) HR sensor. (**b**) ABPSys sensor. (**c**) ABPMean sensor. (**d**) pulse sensor. (**e**) RESP sensor. (**f**) SpO2 sensor. (**g**) ABPDias sensor.

To evaluate the ability of the proposed model to detect point anomalies at every data point in the datasets, the loss rate metric is selected because the datasets are not labeled. Figure 9 shows the loss rate for each physiological sensor in the datasets. 

In Figure 9a,c,g the spike in the loss was showing an unusual behavior at 0.2 and 0.3 loss rates because this is the rate of normal behavior change, which means it changes more than expected. As noted, the number of iterations at 15 and 25 did not fit with the dataset. Therefore, the best scenario for iteration number was at epochs = 30. Hence, it can be concluded that when the number of iterations increases the spike loss decreases and the model is improved. In Figure 9a,d the data loss faults were showing unusual behavior at a 0.3 loss rate. The reason might be the exhibits sensing faults in that period. In Figure 9e,f there are different loss rates. For example, in Figure 9e the loss was high at 0.4, while in Figure 9f the loss was low at 0.08 which refers to the large or small number of anomalous instances, respectively. 

It can be concluded that the number of iterations plays an important role in decreasing the loss rates. In addition, there is an inverse correlation between the loss and the number of repetitions. With an increase in the number of repetitions, the loss rates decrease. Moreover, it can be concluded that the reason for the appearance of the spikes is the nature of the data itself such that the sensors containing more anomalies have more spikes. Thus, the performance of the model was as expected because the structure of ConvLSTM is designed to handle point anomalies issues.

### 4.3. Contextual Anomaly Detection Result and Analysis

To investigate the contextual anomaly detection results, we need to clarify the correlation principle that plays a great role here. Two types of correlations are temporal correlations that exist between each sensor reading and the spatial correlations that exist among the different sensors.

**Figure 10:**
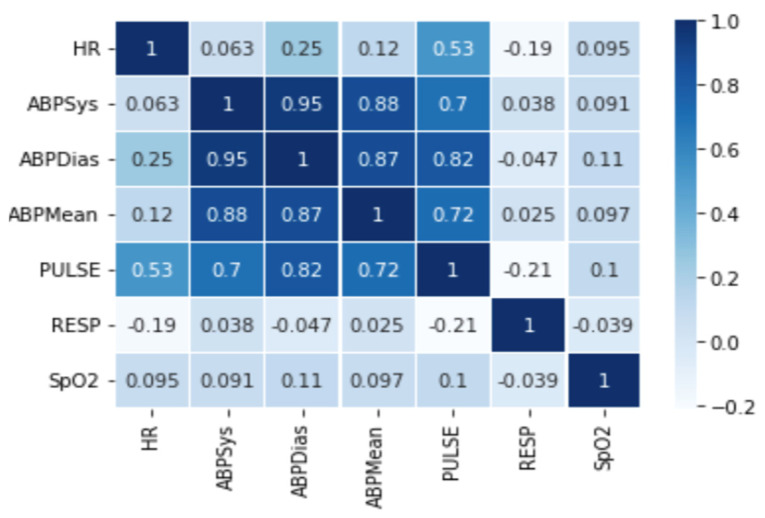
Correlations between physiological data.

The contextual anomaly detection approach uses the context principle, which depends on the correlations between sensor readings and measures how these sensors related to each other. For example, +1 refers to a complete positive correlation and +0.8 refers to a strong positive correlation. Similarly, +0.6 refers to moderate positive correlation, whereas 0 means no correlation. When the values go below zero, it means that a negative correlation exists. For example, −0.6 refers to moderate negative correlation and so on. Figure 10 shows the correlations between all physiological data in the MIMIC dataset. It indicates that there exist various degrees of correlations between the sensors in the dataset, such that ABPSys strongly correlates with ABPDias, ABPMean, and moderately with a pulse. Such a relationship is useful when detecting context anomalies that cannot be detected in a separate context.

Contextual anomaly detection allows distinguishing between physiological anomalies and sensor faults. The output of the point anomaly detector (discussed in Section 4.2.1) with correlation acts as input to the contextual anomaly detector. If different sensors in a collection of associated sensors exhibit point anomalies at the same time, it is most likely a real medical condition. If the value of one sensor in a linked set is abnormal while the other is not, it could be a sensor fault, which could result in a false alarm if it is not properly handled.

To verify the performance of the proposed model, several performance measures are used, which are accuracy, loss, recall, precision, F1-score, and the execution time. The model was verified using 4 datasets (4 subjects) with different size and correlation ratios (a sample with 0.25 correlation rate as in ABPDias and HR, a sample with 0.95 correlation rate as in ABPDias and ABPSys, and a sample with full correlation in the dataset). As shown in Table 6, the proposed model performs well with all 4 subjects in terms of accuracy, recall, precision, and F1-score, where the results ranged between 98% to 100% on all data of different sizes. However, different values of loss rates (high and low) and also different execution times were reported with the 4 subjects. The reason is due to the nature of the data itself, i.e., in Subject 3, the majority of the features (physiological parameters) in the data contained anomalies, which resulted in higher loss rates than usual.

Similarly, based on the correlation relationships between ABPDias and ABPSys sensors, the performance of the proposed model on the same 4 subjects is reported in Table 7. It is clearly shown that on all subjects, better results in terms of accuracy, loss, recall, precision, and F1-score are reported. In terms of execution time, Subject 4 took a bit longer due to its large size.

Physiological data are important indicators to assess the condition of the entire human body. From this standpoint, considering all correlation relationships (whether strong or poor) is also important. Therefore, the proposed model was also evaluated on several subjects, taking into account all the correlation relationships in the data. Table 8 shows that both subject 2 and subject 3 achieved excellent results in terms of accuracy, loss, recall, precision, F1-score and execution time. However, subject 1 and subject 4 reported a slight decrease in accuracy compared to subject 2 and subject 3. This is due to the nature of the data that constitutes a large number of anomalies compared to subjects 2 and 3. 

The proposed model was able to detect the anomalies based on ConvLSTM with a context anomaly score. In the experiment, to test the efficacy and robustness of the proposed model, the data were collected from 4 different subjects with different data sizes. Clearly shown in Table 8, the results of the proposed model made a significant improvement in terms of loss rate, accuracy, recall, precision, F1-score, and time. All the subjects achieved the same execution time (the 60 s). In Subjects 1 and 4, the accuracy and the F1-score reported around 97% and 96% respectively, while the recall and precision achieved 94% and 100% with low loss rates. 

Similarly, for Subjects 2 and 3, the proposed model achieved accuracy and the F1-score of 99%, while a recall and precision of 99% and 100% were also reported. 

Consequently, the model proved that there is an inverse relationship between loss rate and accuracy—when the loss rate has decreased the accuracy is increased. In addition, there is a positive relationship between accuracy, recall, precision, and F1-score—when the accuracy is increased, the recall, precision, and F1-score are also increased. Overall, the proposed model obtained high accuracy and low loss such that it produces few errors on just some amount of data, which is the perfect case. In addition, the model can detect anomalies even in light of big data with the dynamic context changes of WBAN and different dataset sizes and various conditions achieved with fewer time constraints.

## 5. Comparison with Existing Deep Learning and Machine Learning Techniques

Comparative experiments have been conducted with the best and the latest candidates of deep learning and machine learning models in the following subsections. The parameters of the deep learning methods used for comparison are presented in Table 9.

For machine learning methods used in the comparison, a multiclass SVM with linear kernel function, a multiple linear regression, a decision tree with gini criterion, and a random forest with a penalty of 12 was used. For all models, the dataset is split into 70:30 for train and test.

A. Deep Learning Techniques

The proposed model in this paper uses a hybrid model that integrates convolutional and LSTM techniques. This section compares the performance of both convolutional and LSTM separately with the proposed model. As shown in Table 10, the proposed model achieved the best results over convolutional and LSTM, while the convolutional model obtained the lowest accuracy, recall, precision, F1-score, and high loss with less execution time. Likewise, the LSTM model obtained higher accuracy, recall, precision, F1- score, and higher loss with a long execution time. While the hybrid takes the benefits from both LSTM and convolutional, it achieved good results in terms of accuracy, recall, precision, and F1-score within less time. In summary, the combination between the convolutional and the LSTM makes the proposed model more accurate and faster in detecting anomalies in time-series data. Due to the architecture of the convolutional technique that takes benefits from local spatial observations, this allows the model to have fewer weights as some shared data. Consequently, this process makes the model detect the anomaly with high speed. While the cell of LSTM maintains old cell memory at the time, this process helps to understand the behavior of temporal correlations with high accuracy.

B. Machine Learning Techniques

In the literature, most anomaly detection studies for WBAN utilized various machine learning models. Therefore, the proposed model will be compared with some existing machine learning models such as Linear Regression (LR), Decision Tree classifier (DT), Random Forest classifier (RF), and Support Vector Machine (SVM) The same random seed is used in the model parameters to ensure that the training data are split in the same way and that each algorithm is evaluated in the same way. The SVM uses a kernel to transform the input data into the required form; the kernel has been called the kernel trick. The reason behind selecting these algorithms is that they are used for classification purposes and are suitable for the dataset pattern. Table 11 demonstrates the results of these models and it is noted that the decision tree classifier and Random Forest classifier obtained a slightly higher accuracy. Machine learning techniques appear to be not good candidates for detecting the anomaly in light of big data generated by sensors due to the sensitivity to anomalies. For example, in terms of the linear regression technique, the dataset that contains some anomalies can damage the performance of a machine learning model drastically and often lead the model to low accuracy. Any small modification in the dataset can cause a large change in the structure of the decision tree, causing instability. The random forest classifier requires a great deal of time for training as well as much computational power. In addition, the SVM model is not suitable for large datasets. On the contrary, the proposed model, which obtained optimum performance (by improving the accuracy that helps in reducing the false alarm rates) in detecting anomalies, in the context of big data and sensor scalability that makes the healthcare systems more reliable.

To summarize, as shown in Table 11, deep learning is a great option for overcoming the constraints of machine learning models. It works with several processing layers to learn data representation at various abstraction levels. Because deep learning models’ design is dynamic, they can adapt to new patterns. Additionally, it can efficiently analyze large amounts of data in terms of accuracy, memory, and speed. As a result, it outperforms machine learning on massive amounts of data.

## 6. Conclusions and Future Work

IoHT paradigm and WBAN will play a significant role in developing next-generation healthcare applications and services. One primary concern is how to deal with anomalies, as they are imperative in IoHT due to low cost and constrained sensors. Anomalies need to be identified and appropriate action must be taken to maintain the reliability of smart healthcare applications and services. In this paper, the proposed model takes leverage from the ability of LSTM in benefiting from temporal dependencies that exist in sensor readings, and the ability of convolutional neural networks to spatially examine the relationship between more than one sensor. Experiments on the real world dataset for healthcare vital signs called Physionet assure the ability of the proposed model in detecting both point anomalies and contextual anomalies effectively and efficiently. The performance evaluation and comparison with the state-of-art machine and deep learning models showed the superiority of the proposed with an average prediction accuracy of 99% and an F1-score of 98% in 60 s. As a future plan, an investigation of the effect of adversarial attacks on the proposed model will be examined to show the robustness of deep learning techniques against such attacks.

## Figures and Tables

**Table 1:** Analysis of Existing Studies.

Study	Point Anomaly	Contextual Anomaly	Correlation		Thresholds		Faulty Measurements Detection
			Temporal	Spatial	Static	Dynamic	
[3]	✓	✓	X	X	✓	X	✓
[6]	✓	X	X	X	X	X	✓
[7]	✓	✓	✓	✓	X	✓	✓
[8]	✓	✓	X	✓	X	✓	✓
[9]	✓	✓	X	✓	X	✓	✓
[10]	✓	X	✓	✓	✓	X	✓
[11]	✓	X	✓	X	✓	X	✓
[12]	✓	X	X	X	X	X	✓
[13]	✓	X	✓	X	✓	X	✓
[14]	✓	X	X	✓	✓	✓	✓
[15]	✓	✓	X	X	X	✓	✓
[16]	✓	✓	✓	✓	X	✓	✓
[17]	✓	✓	✓	✓	X	✓	✓
[18]	✓	X	X	X	X	X	✓
[19]	✓	X	✓	X	X	✓	✓
[20]	✓	X	X	✓	X	✓	✓
[21]	✓	X	✓	✓	✓	X	✓
[22]	✓	X	✓	X	✓	X	✓
[23]	✓	✓	✓	✓	X	✓	✓
[24]	✓	X	✓	X	X	✓	✓

**Table 2:** Sample of sensor readings for Subject 1 dataset.

*Time and Date*	HR	ABPSys	ABPDias	ABPMean	PULSE	RESP	SpO2
**14:07:00 10/11/15**	77.6	157.4	66.1	100.5	77.9	23	97.4
**14:08:00 10/11/15**	77.3	149.2	62.6	95	77.6	22.2	97
**14:09:00 10/11/15**	76.1	150.5	62.4	95.1	76.8	22.3	97
**14:10:00 10/11/15**	73	158.4	65.4	99.8	74.3	22.2	97.4
**14:11:00 10/11/15**	75.6	152.4	63.3	96.7	76.4	22.4	97.5
**14:12:00 10/11/15**	75	154.3	63.4	97.1	75.4	22.2	97.5
**14:13:00 10/11/15**	75.2	150.3	62.1	94.7	76.7	22.1	97.6

**Table 3:** Definition and description of the variables used in the LSTM model [32].

Variable	Definition and Description
$W_{f}$	the weights matrices of forget gate ($f_{t}$)
$h_{t-1}$	The output from the cell at time t−1
$X_{t}$	the current input at time t
$B_{f}$	The bias in the forget gate
$W_{i}$	the weights matrices of input gate ($i_{t}$)
$B_{i}$	The bias in the input gate
$W_{o}$	the weights matrices of output gate ($o_{t}$)
$B_{o}$	The bias in the output gate
$c_{t}$	The cell state at time t
${c^{\prime }}_{t}$	The data stored in the new cell state $c_{t}$
$W_{c}$	the weights matrices of the new cell state $c_{t}$
$B_{c}$	The bias in new cell state $c_{t}$
$h_{t}$	The hidden states at sequential time t

**Table 4:** Definition and description of the variables used in Conv-LSTM model [36].

Variable	Definition and Description
$X_{t}$	The input tensor at time t
$H_{t-1}$	The output tensor from the cell at time t−1
$h_{t}$	The output tensor from the cell at time t
$C_{t-1}$	The cell state at time t−1
${c^{\prime }}_{t}$	The data that stored in the new cell state $C_{t}$
$c_{t}$	The cell state at time t
$f_{t}$	Output of the forget gate and it controls the data that is forgotten in the old cell state $C_{t-1}$
$i_{t}$	Output of the input gate, it controls how much of the data ${c^{\prime }}_{t}$ will be stored in the new cell state $c_{t}$
$o_{t}$	Output of the output gate, it controls the data that is output $h_{t}$ from the cell.
$W_{\mathrm{xf}}$	The convolution kernel used to the input tensor $X_{t}$ in the forget gate
$W_{\mathrm{xi}}$	The convolution kernel used to the input tensor $X_{t}$ in the input gate.
$W_{\mathrm{xc}}$	The convolution kernel used to the input tensor $X_{t}$ for create data ${c^{\prime }}_{t}$ that will be stored in the new cell state $c_{t}$.
$W_{\mathrm{xo}}$	The convolution kernel used to the input tensor $X_{t}$ in the output gate
$W_{\mathrm{hf}}$	The convolution kernels used to the input tensor $H_{t-1}$ in the forget gate.
$W_{\mathrm{hi}}$	The convolution kernels used to the input tensor $H_{t-1}$ in the input gate
$W_{\mathrm{hc}}$	The convolution kernel used to the input tensor $H_{t-1}$ for create the data ${c^{\prime }}_{t}$ that will be stored in the new cell state $c_{t}$.
$W_{\mathrm{ho}}$	The convolution kernels used to the input tensor $H_{t-1}$ in the output gate.
$W_{\mathrm{cf}}$	The weight that is used to the old cell state $C_{t-1}$ in the forget gate.
$W_{\mathrm{ci}}$	The weight that is used to the old cell state $C_{t-1}$ in the input gate
$W_{\mathrm{co}}$	The weight that is used to the new cell state $c_{t}$ in the output gate.
$B_{f}$	The bias in the forget gate.
$B_{i}$	The bias in the input gate
$B_{c}$	The bias for creating the data ${c^{\prime }}_{t}$ that will be stored in the new cell state $c_{t}$
$B_{o}$	The bias in the output gate.

**Table 5:** Physiological data normal and abnormal ranges [23].

Physiological Parameter	Normal Range	Abnormal Range
Heart rate	60–100	<60 and >100
Pulse rate	60–100	<60 and >100
Reparation rate	12–30	<12 and >30
SpO2	95–100	<95 and >100
ABPDias	80–120	<80 and >120
ABPSys	90–120	<90 and >120
ABPMean	70–100	<60 and >110

**Table 6:** Performance evaluation (different datasets with correlated ABPDias with HR sensors).

Subject No.	Size	Accuracy (%)	Loss (%)	Recall (%)	Precision (%)	F1-Score (%)	Time (s)
Subject 1	1.02 MB	99.30%	0.0018%	100%	98.25%	99.12%	86 s
Subject 2	467 KB	99.59%	0.0063%	98.89%	99.58%	99.46%	48 s
Subject 3	885 KB	99.89%	0.0023%	100%	100%	99.88%	63 s
Subject 4	1.03 MB	99.94%	0.0008%	100%	99.93%	99.96%	96 s

**Table 7:** Performance evaluation (different datasets with correlated ABPDias with ABPSys sensors).

Subject No.	Accuracy (%)	Loss (%)	Recall (%)	Precision (%)	F1-Score (%)	Time (s)
Subject 1	99.90%	0.0066%	100%	99.94%	99.92%	47 s
Subject 2	99.97%	0.0053%	100%	100%	99.98%	87 s
Subject 3	99.96%	0.0017%	100%	99.42%	99.95%	89 s
Subject 4	99.94%	0.0036%	100%	100%	99.77%	145 s

**Table 8:** Performance Evaluation (Different Datasets with Full Correlation).

Subject No.	Accuracy (%)	Loss (%)	Recall (%)	Precision (%)	F1-Score (%)	Time (s)
Subject 1	97.59%	0.1131%	99.40%	95.56%	97.59%	60 s
Subject 2	99.91%	0.0112%	99.93%	99.87%	99.90%	60 s
Subject 3	99.97%	0.0022%	100%	99.86%	99.96%	60 s
Subject 4	97.69%	0. 028%	100%	94.05%	96.93%	60 s

**Table 9:** Deep learning models’ parameters.

Parameters	LSTM Value	CNN Value
Language	Python	Python
Libraries	Pandas, Numpy, Scikitlearn, Matplotlib and Keras	Pandas, Numpy, Scikitlearn, Matplotlib and Keras
Train set	70%	70%
Test set	30%	30%
Input Layer	4	4
Activation Functions	Rectified Linear Unit (ReLu), and sigmoid	Rectified Linear Unit (ReLu)
Dense Layer	2	2
Dropout	0.20	0.20
Optimizer	Adam	Adam
Number of Epochs	30	30
Batch size	72	72

**Table 10:** Comparison with deep learning models.

Model	Subject	Accuracy (%)	Loss (%)	Recall (%)	Precision (%)	F1-Score (%)	Time (s)
Convolutional	Subject 1	46.14%	0.6602%	100%	46.14%	63.14%	21 s
	Subject 2	68.30%	0.570%	100%	41%	58%	73 s
	Subject 3	70.80%	0.5678%	99.93%	44.56%	61.63%	62 s
	Subject 4	36.53%	0.5896%	100%	36.53%	53.51%	40 s
LSTM	Subject 1	76.25%	0.3649%	99.74%	46.07%	63.03%	90 s
	Subject 2	95.89%	0.1252%	98.63%	41.12%	58.23%	317 s
	Subject 3	94.45%	0.118%	100%	44.57%	61.66%	506 s
	Subject 4	86.73%	0.279%	99.14%	36.36%	53.20%	480 s
CNN-LSTM	Subject 1	97.59%	0.1131%	99.40%	95.56%	97.59%	60 s
	Subject 2	99.97%	0.0022%	100%	99.86%	99.96%	60 s
	Subject 3	99.91%	0.0012%	99.93%	99.87%	99.90%	60 s
	Subject 4	97.69%	0. 028%	100%	94.05%	96.93%	60 s

**Table 11:** Comparison with machine learning models.

Model	Subject	Accuracy (%)	Recall (%)	Precision (%)	F1-Score (%)
LR	Subject 1	95.50%	94%	97%	96%
	Subject 2	96.17%	99%	94%	97%
	Subject 3	93.33%	95%	93%	94%
	Subject 4	88.78%	87%	96%	92%
DT	Subject 1	99.92%	63%	71%	67%
	Subject 2	100%	100%	83%	91%
	Subject 3	99.88%	89%	99%	94%
	Subject 4	100%	92%	76%	83%
RF	Subject 1	99.92%	94%	97%	96%
	Subject 2	100%	99%	94%	97%
	Subject 3	99.91%	93%	95%	94%
	Subject 4	100%	66%	94%	78%
SVM	Subject 1	61.82%	71%	63%	67%
	Subject 2	87.69%	100%	83%	91%
	Subject 3	93.04%	99%	89%	94%
	Subject 4	65.70%	66%	94%	78%
Proposed Model	Subject 1	97.59%	99.40%	95.56%	97.59%
	Subject 2	99.91%	99.93%	99.87%	99.90%
	Subject 3	99.97%	100%	99.86%	99.96%
	Subject 4	97.69%	100%	94.05%	96.93%
